# Thickness Optimization of Thin-Film Tandem Organic Solar Cell

**DOI:** 10.3390/mi12050518

**Published:** 2021-05-05

**Authors:** Kamran Ali Bangash, Syed Asfandyar Ali Kazmi, Waqas Farooq, Saba Ayub, Muhammad Ali Musarat, Wesam Salah Alaloul, Muhammad Faisal Javed, Amir Mosavi

**Affiliations:** 1 Department of Electrical Engineering, Sarhad University of Science and Information technology, Peshawar 25000, Pakistan; engrkamran199@gmail.com (K.A.B.); asfandyaralikazmi@gmail.com (S.A.A.K.); waqasfarooq.ee@gmail.com (W.F.); 2 Department of Fundamental and Applied Sciences, Universiti Teknologi PETRONAS, Bandar Seri Iskandar, Tronoh 32610, Perak, Malaysia; saba_20000009@utp.edu.my; 3 Department of Civil and Environmental Engineering, Universiti Teknologi PETRONAS, Bandar Seri Iskandar, Tronoh 32610, Perak, Malaysia; wesam.alaloul@utp.edu.my; 4 Department of Civil Engineering, COMSATS University Islamabad Abbottabad Campus, Abbottabad 22060, Pakistan; arbabfaisal@cuiatd.edu.pk; 5 Faculty of Civil Engineering, Technische Universität Dresden, 01069 Dresden, Germany; 6 Department of Informatics, J. Selye University, 94501 Komarno, Slovakia; 7 Information Systems, University of Siegen, Kohlbettstraße 15, 57072 Siegen, Germany; 8 John von Neumann Faculty of Informatics, Obuda University, 1034 Budapest, Hungary

**Keywords:** organic solar cells, triple junction, solar energy, tandem, renewable energy, energy harvesting, temperature, photovoltaic, micromachines, thin-film

## Abstract

The polymer solar cells also known as organic solar cells (OSCs) have drawn attention due to their cynosure in industrial manufacturing because of their promising properties such as low weight, highly flexible, and low-cost production. However, low *η* restricts the utilization of OSCs for potential applications such as low-cost energy harvesting devices. In this paper, OSCs structure based on a triple-junction tandem scheme is reported with three different absorber materials to enhance the absorption of photons which in turn improves the *η*, as well as its correlating performance parameters. The investigated structure gives the higher value of *η* = 14.33% with *J_sc_* = 16.87 (mA/m^2^), *V_oc_* = 1.0 (V), and *FF* = 84.97% by utilizing a stack of three different absorber layers with different band energies. The proposed structure was tested under 1.5 (AM) with 1 sun (W/m^2^). The impact of the top, middle, and bottom subcells’ thickness on *η* was analyzed with a terse to find the optimum thickness for three subcells to extract high *η*. The optimized structure was then tested with different electrode combinations, and the highest *η* was recorded with FTO/Ag. Moreover, the effect of upsurge temperature was also demonstrated on the investigated schematic, and it was observed that the upsurge temperature affects the photovoltaic (PV) parameters of the optimized cell and *η* decreases from 14.33% to 11.40% when the temperature of the device rises from 300 to 400 K.

## 1. Introduction

Organic solar cells (OSCs) have been a point of attention and main consideration for many research communities over the past few decades due to their countless advantages such as light weight, thin size, low manufacturing cost, and semi-transparency. To date, the extracted conversion efficiency for multi-layer solar cells based on absorption material has not much exceeded as collating to inorganic solar cells [1]. The recorded *η* is, however, slightly more than the single-layer-based OSCs [2], which indicates that there is still a great chance for improving the schematic of tandem organic solar cells (TOSCs). For better configuration of TOSCs, the optimal feature of bandgap energies should be the main consideration that needs to be optimized for extracting high conversion efficiency. The theoretical view for TOSCs cells has the potential to counter the double junction drawbacks in terms of improved PV parameters, i.e., *J_s_*, *FF*, *V_oc_*, and *η* [3]. For TOSCs, the differences in the bandgap *E_g_* values of the top, middle, and bottom subcells absorber layers would be adequate and cause maximum chances for each subcell to generate a large number of carriers [4]. For example, the multi-junction thin-film solar cells have an optimal configuration to obtain high current which improves the overall *η* of the cell, and thus tandem features top absorber layer with wide bandgap, medium absorber layer with intermediate bandgap, and bottom absorber layer with a low bandgap. Therefore, the configuration of the tandem cells formed on bandgap becomes *E_g_*_1_ > *E_g_*_2_ > *E_g_*_3_ as suggested by [5,6,7]. This optimal scheme of bandgap cannot be implemented directly to OSCs due to a lack of donor materials having band energies as small as 1 eV [8]. However, when the material has a low bandgap such as 1 eV, it will give rise to several issues such as the low value of (*V_oc_*) and lowest unoccupied molecular orbital (LUMO) which results in low efficiency of charge separation [9].

To cover a broad spectrum of radiation with enhanced absorption, absorber materials with a large band need to be produced and designed first or various narrow or low bandgap absorbers have to be stacked in tandem multi-junction [9]. In TOSCs, when two non-overlapping absorption spectra are stacked, a broad range of absorption spectra including visible and IR range can be utilized [10]. Depending upon the absorber materials used for absorption different approaches have been demonstrated. Generally, tandem solar cells can be categorized into three types as described by Peumans et al. [10]. Here, it is important to mention that structures like these are complex to realize via solution processing due to the dissolution of orthogonal which cannot be circumvented [11]. In context to the thin-film solar cell, thickness optimization is one of the key parameters to extract high conversion efficiency because the thicker the layer, the higher the cost. Thus, to reduce the cost of the cell, the optimization of the active layer is the hotspot point. Moreover, if the optimization is not performed, the recombination losses occur and result in the low performance of the cell. Here, it is important to mention that utilization of the optimized material along with the combination of the compatible layer is needed to obtain high-performance electrical photovoltaic parameters. Structures without optimization and with unmatched stacking layers result in low absorption of photons, which in turn delivers low performance because such structure suffers from dominant losses. The main objective of this study was to analyze the impact of thickness optimization on the tandem organic solar cell to obtain improved efficiency.

Herein, we investigated a tri-layer tandem OSC at an optimized temperature of 300 K, which is engineered in such a way to maximize the output photocurrent incorporating with bandgap energies. The schematic of the investigated structure consists of the three absorber layers in which top material has wide bandgap *E_g_*_1_, middle absorber layer has medium bandgap *E_g_*_2_, and bottom absorber layer has low bandgap *E_g_*_3_, such as *E_g_*_1_ > *E_g_*_2_ > *E_g_*_3_. The investigated structure is Glass/FTO/TiO_2_/first absorber/MoO_3_/TiO_2_/second absorber/WO_3_/MoO_3_/TiO_2_/third absorber/WO_3_/Ag. 

## 2. Device Modeling and Working

In this investigation, the presented schematic was framed with thirteen effective layers in which the top electrode is composed of transparent conductive oxide, i.e., FTO as a front electrode because it has dual nature as it has high transparency in the visible spectrum and also fetches carriers when used as an electrode [12]. In addition to this, it has low sheet resistance and high work function. After the FTO, TiO_2_ is introduced as a window layer (WL) because of its high transmission, low resistance, and lower cost as compared to other window layer materials. After WL, the first top absorber material (P3HT: PCBM) with a high bandgap is stacked with variable thickness. After the first absorber layer, MoO_3_ is introduced as a hole transporting layer (HTL) [13] and is stacked below the P3HT: PCBM layer. Next, again, TiO2 is placed below the MoO3 layer, thus making a tunnel junction and connecting layers with the next subcells. After the tunnel junction, a second absorber layer (PTB7: PCBM) with an intermediate bandgap is introduced for capturing those photons that were left unabsorbed in the first absorber layer. Right below the second absorber material, an excellent hole extracting layer (HEL), i.e., tungsten trioxide (WO_3_), is introduced because of its matchless properties such as hole transport carrier, non-toxicity, easy evaporation, and low cost [14,15]. After HEL, again MoO3 and TiO2 are placed for making the second tunnel junction for the next subcell. After the second tunnel junction, a low bandgap absorber material PDTS-DFTTBT: PCBM is introduced. After the third absorber layer, again tungsten trioxide (WO_3_) is placed because it has a high work function and can boost the hole transport and collection at the anode interference (WO_3_/Ag). Moreover, it reduces the series and contact resistance. 

All the electrical simulations were performed in General-Purpose Photovoltaic Device Model (GPVDM) software. In this study, the electrical model of the cell is considered. The *J_sc_* of the device is obtained by the photocurrent densities. The incident radiation that produces photocurrent density can be written as [16,17].

(1) $$J_{phl}\left(V\right)=\int_{0}^{\infty }\left\{J_{p}\left(\lambda ,V\right)+J_{n}\left(\lambda ,V\right)\right\}d\lambda$$

where for holes and electrons photocurrent density is represented as $J_{p}\left(\lambda ,V\right)$ and $J_{n}\left(\lambda ,V\right)$ in Equation (1). 

The photocurrent density for electron solving is expressed as:

(2) $$J_{n}\left(\lambda ,V\right)=\frac{e}{W}\left\{\mu_{n}F\int_{0}^{W}\delta_{n}dx-D_{n}\int_{0}^{W}\frac{\partial \delta_{n}}{\partial x}dx\right\}$$

Similarly, for electrons, the photocurrent densities become:

(3) $$J_{p}\left(\lambda ,V\right)=\frac{e}{W}\left\{\mu_{p}F\int_{0}^{W}\delta_{p}dx-D_{p}\int_{0}^{W}\frac{\partial \delta_{p}}{\partial x}dx\right\}$$

The continuity equation is described as:

(4) $$\frac{\partial }{\partial t}\left(\delta_{n}\right)=\mu_{n}F\frac{\partial }{\partial x}\left(\delta_{n}\right)+D_{n}\frac{\partial^{2}\delta_{n}}{\partial x}+Ge^{-X\alpha \left(\lambda \right)}-\frac{\delta_{n}}{\tau_{n}}=0$$

where $\delta_{n}$ is the concentration of generated electrons, $\mu_{n}$ and $D_{n}$ are the electron mobility and diffusion, *G* is the optical generation rate of the carrier, and *F* in the equation shows the electric field in the cell. 

The *V_oc_* is given by:

(5) $$V_{oc}=\frac{nkT}{q}\times ln\left(\frac{J_{pki}}{J_{0}}+1\right)$$

where k is the Boltzmann constant, n is the ideality factor, q is the electron charge density, $J_{0}$ is the saturation current density, and T is the temperature.

The Fill factor (*FF*) of the optimized cell can be calculated as:

(6) $$FF=\frac{J_{mp}V_{mp}}{J_{sc}V_{oc}}$$

300 K is the optimized temperature, which is kept constant throughout the simulation.

## 3. Results and Discussion

**Figure 1:**
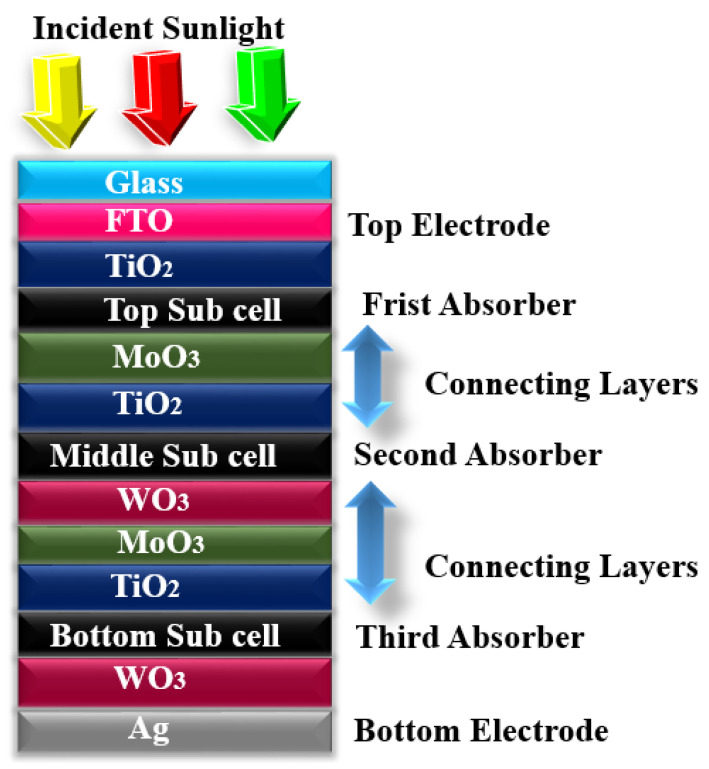
Configuration of a proposed structure.

In this investigation, tandem structures are analyzed based on three junctions as shown in Figure 1, the follow-up strategy for configuring the three junctions is to absorb the non-absorbed photons from single- and double-layer junctions. Thickness is optimized for the top, middle, and bottom subcell layers to extract high *η*. The influence of top, middle, and bottom subcell thickness on the performance parameters, i.e., *V_oc_*, *J_sc_*, *FF,* and *η*, are studied. Three different absorber layers were utilized in such a way that P3HT: PCBM is used as a top active layer having coefficient of absorption α = 52,925 cm^−1^, PTB7: PCBM for second active layer having coefficient absorption α = 67,237 cm^−1^ [18] and PDTS-DFTTBT: PCBM absorber material for bottom subcell because its bandgap is narrower than P3HT: PCBM and PTB7:PCBM.

### 3.1. Investigation on Top Subcell

**Figure 2:**
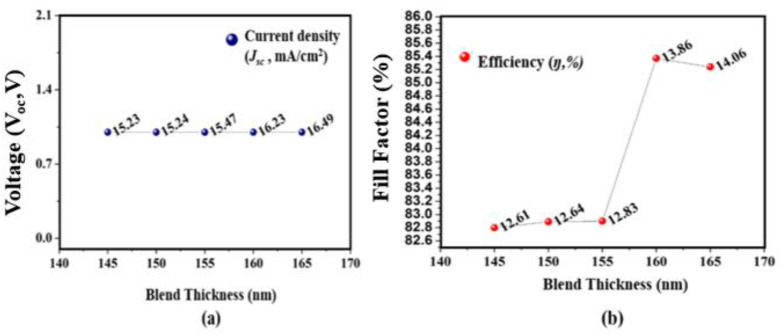
Effect of top active absorber layer thickness on photovoltaic parameters. (**a**) *V_oc_* and *J_sc_*; (**b**) *FF* and *η*.

The proposed structure is firstly analyzed by altering the thickness of the top absorber layer, as these layers perform a key role in the performance of the device. The optimization of the cell thickness is the critical parameter as there is a mismatch of the current when dealing with tandem structures. The thickness of the top layer is altered between 145 and 165 nm while keeping the middle and bottom subcell thickness fixed at 120 and 90 nm. By growing the thickness of the cell from 145 to 165 nm, the *J_sc_* rises from 15.23 to 16.49 (mA/cm^2^) as shown in Figure 2a. This rise in *J_sc_* is because when the thickness of the cell height, the area of the absorber, increases, a large amount of sunlight which consists of photons is absorbed and results in a greater number of excitons, and thus the *J_sc_* increases. Here, the *V_oc_* is not much affected while varying the absorber thickness. Figure 2b shows the influence of absorber layer thickness on *FF* and η. The *J_sc_* and *V_oc_* are directly linked to *FF* and *η*, as a result when the thickness increases, the *FF* and *η* also increase. The highest recorded *η* while varying the top layer thickness is 14.06% at 165 nm and *FF* = 85.24%.

### 3.2. Investigation on Middle Subcell 

**Figure 3:**
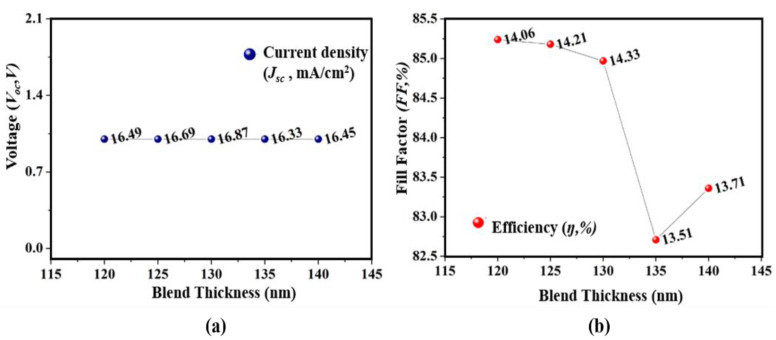
Effect of second absorber layer thickness on photovoltaic parameters. (**a**) *V_oc_* and *J_sc_*; (**b**) *FF* and *η*.

After analyzing the top subcell thickness, the middle subcell thickness is altered from 120 to 140 nm while keeping the top subcell and bottom subcell thickness fixed at 165 and 90 nm. It is shown in Figure 3a that the middle subcell thickness is found to be optimized at 130 nm because when the thickness of the subcell increases from 130 nm, the *J_sc_* tends to decrease, and the decrease in the *J_sc_* value is because of unmatched carrier generation. At 130 nm, the highest *η* of 14.33% is recorded and *FF* is found to be at 84.97% as shown in Figure 3b. This suggests that there is a need to optimize the thickness of the subcell because by modifying the thickness of the subcell, thicknesses can redistribute the uneven photon absorptions and cause matched photocurrents [19,20]. 

### 3.3. Investigation on Bottom Subcell

**Figure 4:**
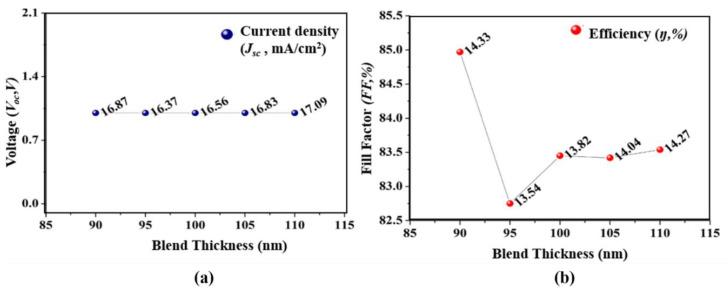
Effect of third absorber layer thickness on photovoltaic parameters. (**a**) *V_oc_* and *J_sc_*; (**b**) *FF* and *η*.

Next, the second absorber thickness of the subcell is altered from 90 to 110 nm while keeping the remaining two subcell thickness fixed at 165 and 130 nm, and it is found that the adequate height for the third absorber layer (bottom subcell) is 90 nm. As shown in Figure 4a, the *J_sc_* at 90 nm was found to be highest among the varied thickness, and at this thickness, the *η* recorded to be at 14.33% which is the highest among the other varied thicknesses. The reported results provide a smooth agreement with Lambert–Beer’s law that can be stated as:

(7) A(λ)=e(λ)lc

**Figure 5:**
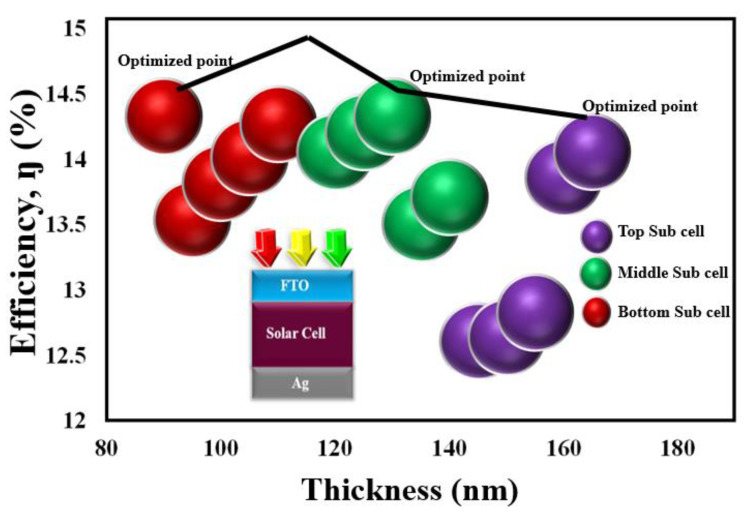
Optimized thickness for top, middle, and bottom Subcell.

**Figure 6:**
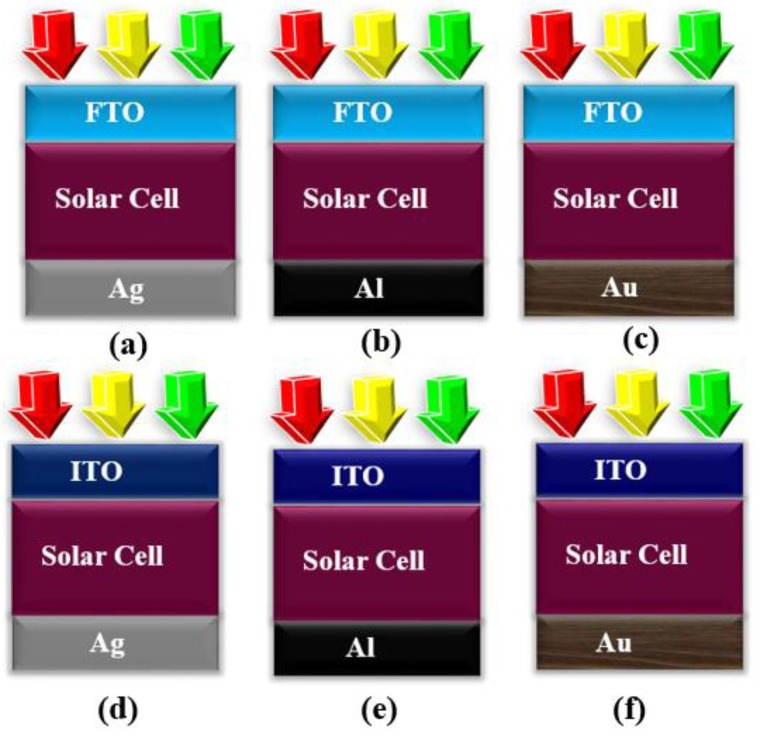
Different investigated electrode configuration: (**a**) FTO/Ag, (**b**) FTO/Al, (**c**) FTO/Au, (**d**) ITO/Ag, (**e**) ITO/Al, and (**f**) ITO/Au.

The coefficient of absorbance wavelength and carriers path length in (7) is symbolized as e(λ) and l. Thus, from the obtained results, the thickness of the top, middle, and bottom subcell was optimized to be at 165 nm, 130 nm, and 90 nm as summarized in Figure 5. Further, an increase in the thickness results in a decrease in performance parameters because of inefficient charges. After optimizing the thickness for the top, middle, and bottom subcell, we tested different electrode configurations with the proposed structure as depicted in Figure 6, and their extracted parameters are summarized in Table 1. The different scheme for electrode configuration with the optimized cell helps in selecting the best material for the top and bottom electrode. The investigation on the electrode configuration suggests that the selection of FTO/Ag for the top and bottom electrode holds 0.32%, 0.45%, 1.02%, 1.3%, and 1.35% more efficiency as compared to FTO/Al, FTO/Au, ITO/Al, ITO/Ag, and ITO/Au, respectively. 

**Figure 7:**
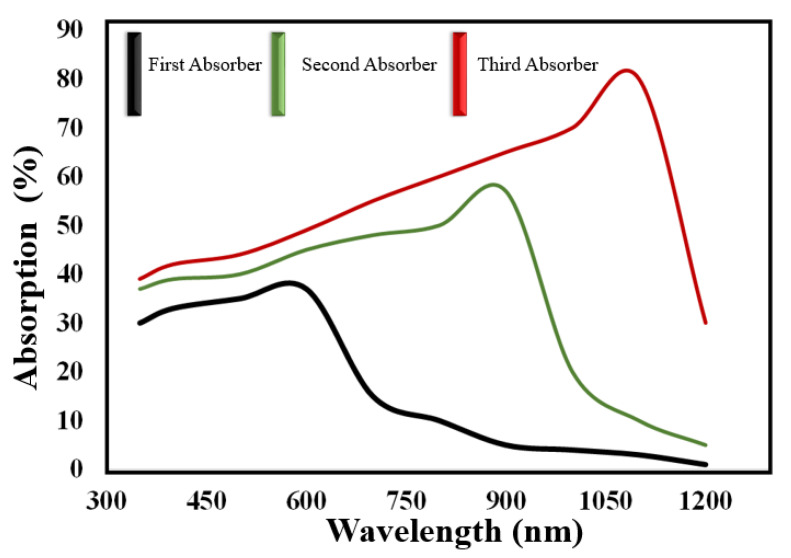
Absorption spectrum coverage against the wavelength.

The absorption spectrum range of the investigated cell is displayed in Figure 7, which suggests that the utilization of the three absorber layers helps to cover a broad spectrum range of wavelength and covers ~80% of the absorption.

### 3.4. High-Temperature Analysis

High temperature degrades the stability and performance of the cell. Here in this section, the influence of high temperature on the investigated structure is tested. The temperature dependence with the bandgap (*E_g_*) of the absorber layer by the Varshni equation can be presented as [17]:

(8) $$E_{g}\left(T\right)=E_{g}\left(0\right)-\frac{\propto T^{2}}{\left(T+\beta \right)}$$

The generated *J_sc_* rely on the spectrum of given solar irradiance, and at each temperature, the *J_sc_* is calculated by (9) and is given by [17]:

(9) $$J_{sc}\int_{hv=E_{g}}^{\infty }\frac{dN_{ph}}{dhv}d\left(hv\right)$$

**Figure 8:**
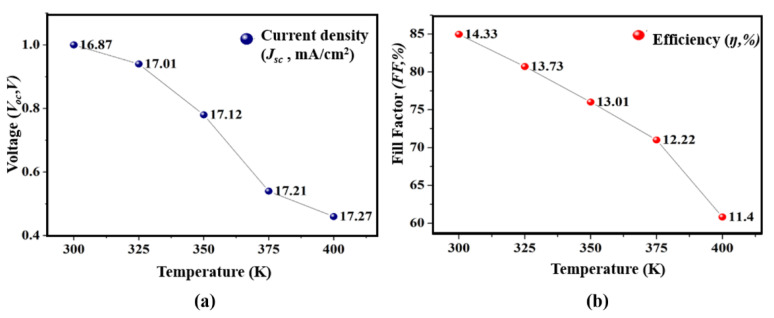
Influence of temperature on photovoltaic parameters. (**a**) *V_oc_* and *J_sc_*; (**b**) *FF* and *η*.

In Figure 8a, it is visible that the *V_oc_* is greatly dropped from 1.00 (V) to 0.45(V). The observed drop in the *V_oc_* values is due to the reverse saturation current which changes with the temperature [21]. There is a slight increase in the *J_sc_* values, which does not contribute to the improvement. The small rise in *J_sc_* is due to reduction in the size of the bandgap *E_g_* which reduces with high temperature, and as a result, more photons get absorbed and a slight increase occurs [21]. The *FF* and *η* also drop with the rise in temperature intensity. In this investigation, the *η* drops from 14.33% to 11.40% when the temperature of the device increased from 300–400 K as depicted in Figure 8b.

## 4. Conclusions

In this work, we optimized OSCS based on the tandem design with three different absorber materials (P3HT: PCMB, PTB7: PCMB, and PDTS-DFTTBT: PCBM) in which transition metal oxide layer tungsten trioxide (WO_3_) was also utilized as a hole transporting layer. By optimizing the thickness of the active layers, we obtained a *η* of 14.33% at 300 K. Moreover, the influence of high temperature was also tested, and it was observed that the upsurge temperature has greatly affected the PV parameters. These results revive a new avenue for many researchers in the field of photovoltaic notably in the field of thin-film solar cell technologies.

## Figures and Tables

**Table 1:** Extracted performance parameters with different electrode configurations.

Electrodes Combination	*Jsc* (mA/cm^2^)	*FF*%	*η*%
FTO/Ag	16.87	84.97	14.33
FTO/Al	15.64	81.02	14.01
FTO/Au	15.55	79.23	13.88
ITO/Al	14.84	78.90	13.31
ITO/Ag	14.61	77.02	13.03
ITO/Au	14.57	76.95	12.98

## Data Availability

All the data are available within this manuscript.
